# Recurrent Spontaneous Pneumomediastinum in an Adolescent Competitive Swimmer: A Case Report

**DOI:** 10.7759/cureus.100289

**Published:** 2025-12-28

**Authors:** Riho Tanimura, Gaku Murakami, Manami Ueshima, Hiroshi Ohuchi

**Affiliations:** 1 Department of Pediatrics, Kameda Medical Center, Kamogawa, JPN; 2 Department of Sports Medicine, Kameda Medical Center, Kamogawa, JPN

**Keywords:** adolescent, competitive swim, recurrent pneumomediastinum, respiratory rehabilitation, return to sport, spontaneous pneumomediastinum

## Abstract

Spontaneous pneumomediastinum (SPM) is a rare, usually self-limited condition in children and adolescents. Sports-related cases are generally considered benign, with early return to play often recommended. However, evidence to guide management in athletes who rely heavily on breath-holding, such as competitive swimmers, is limited. We report a 14-year-old male competitive swimmer who experienced three episodes of SPM over seven months. His initial presentation involved acute anterior chest pain with stable vital signs and mediastinal emphysema on CT. He improved with conservative treatment and returned to full training within one month, avoiding intense breath-holding for two weeks. A second presumptive episode occurred four months later, followed by a third episode three months after that, again with mediastinal emphysema on imaging. We hypothesized that persistent local tissue vulnerability, possibly incomplete healing of prior microinjury, together with rapid resumption of breath-hold training, contributed to recurrence. After the third episode, all training was restricted for two months, during which he completed supervised stretching and diaphragmatic breathing rehabilitation, followed by a gradual return to swimming while avoiding prolonged breath-holding. He has remained free of recurrent SPM. This case suggests that athletes who perform intense breath-holding may require more prolonged activity restriction, targeted respiratory rehabilitation, and explicit limitations on breath-holding to reduce the risk of SPM recurrence.

## Introduction

Spontaneous pneumomediastinum (SPM) is a very rare condition in the general population, with an estimated incidence of approximately 0.002% [1]. Its physiopathology was explained by Macklin [2] as a three-step process involving alveolar rupture, gas dissection through the bronchovascular fascia, and pulmonary interstitial dissemination to the mediastinum. Imaging studies can confirm this process. In otherwise healthy adolescents, the condition often results from a sudden increase in intrapleural pressure during activities involving the Valsalva maneuver (e.g., heavy lifting or intense exercise) [3,4]. SPM recurrence is uncommon, with a narrative review reporting an average recurrence rate of 0.98% [5]. Athletic exertion, cough, asthma flares, and recreational drug use are recognized triggers [6,7]. Sports-related spontaneous pneumomediastinum is generally benign and carries an extremely low risk of recurrence [8,9]. However, evidence regarding athlete-specific management, particularly concerning sports cessation and training programs aimed at reducing the risk of recurrence, remains limited. Here, we report a case of SPM with three recurrences in a competitive swimmer and describe our successful strategies for exercise restriction and return-to-sport.

## Case presentation

**Figure 1:**
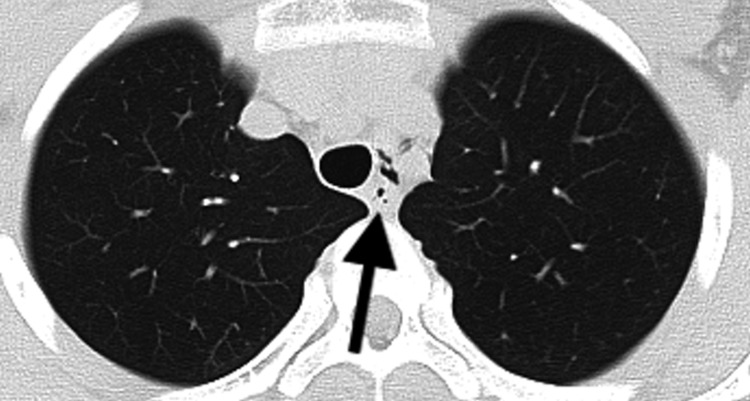
Chest CT images showing pneumomediastinum surrounding the left main bronchus (first episode) CT: computed tomography

A 14-year-old male competitive swimmer presented to the clinic in the morning with acute-onset chest pain that awakened him at approximately 3 a.m. on the day of presentation. He described the pain as a sharp, squeezing sensation in the anterior chest, which worsened with deep inspiration and movement. He reported having experienced occasional mild chest discomfort in the morning for the past six months, particularly after swimming. The current symptom did not improve, prompting him to seek medical evaluation. The patient had a mild pollen allergy, was taking no medications, and had no notable medical or surgical history. The patient denied any history of trauma, asthma, reactive airway disease, or spontaneous pneumothorax. The patient’s vital signs included a blood pressure of 132/77 mmHg, a heart rate of 68 beats per minute, a respiratory rate of 20 breaths per minute, an oxygen saturation of 98% on room air, and a body temperature of 36.5℃. The patient’s height was 162.2 cm; weight, 51.3 kg; and body mass index, 19.5. The patient was in no acute distress. There was no subcutaneous emphysema on his neck, and his breath sounds were clear bilaterally. No cutaneous abnormalities or subcutaneous emphysema were noted. Echocardiography and electrocardiogram showed no remarkable findings. On his chest X-ray, no remarkable lesion was noted. His CT revealed a small amount of mediastinal emphysema extending from the left supraclavicular region to the area surrounding the left main bronchus (Figure 1). No apparent pleural effusion or fluid collection was identified. The patient was admitted to the pediatric service for observation and pain control. Following admission and bed rest, the patient’s pain subsided. In the absence of any radiographic deterioration, discharge occurred on the third hospital day. Physical activity was resumed two weeks later, with gradual increases in intensity. Following discharge, high-intensity breath-holding exercises were avoided. Full activity was achieved one month after initiating exercise.

Four months later, during underwater training, he developed recurrent chest pain that persisted despite rest. The pain worsened with deep inspiration and radiated from the lower ribs toward the throat, prompting him to seek medical care. Chest radiography revealed no remarkable findings. Based on the clinical presentation, we considered this event a probable recurrence of SPM, and the patient was managed conservatively. Because his symptoms were mild and improved rapidly, we deferred repeat CT to limit cumulative radiation exposure. He was discharged on the second hospital day. A plan was made for two weeks of rest following discharge, followed by a gradual resumption of physical activity over the next two weeks.

**Figure 2:**
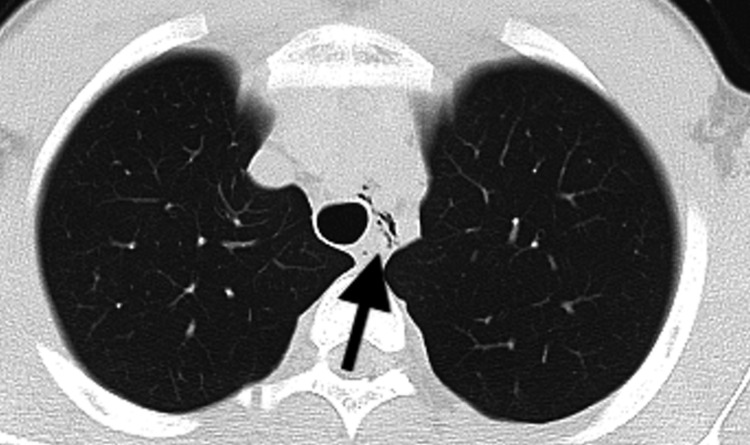
Chest CT images showing pneumomediastinum surrounding the left main bronchus (third episode) CT: computed tomography

The third episode occurred approximately three months later. He developed acute throat pain during swim practice. The pain continued after he returned home. By the following morning, it had worsened and extended to the epigastric region. It was exacerbated by deep inspiration and radiated from the throat to the upper epigastrium. He reported that the symptoms were similar to his prior two episodes, which had also begun with throat pain, and he therefore presented to our emergency department. Chest CT confirmed left-sided mediastinal emphysema, similar to the previous episode (Figure 2). The patient was rehospitalized for four days and subsequently discharged. Possible contributing factors to the recurrence included incomplete healing of the previously injured bronchial tissue and insufficient restriction of breath-holding maneuvers. Following discharge, the patient began attending sessions with a sports trainer at a clinic. Physical activity, including land-based training, was restricted for two months. During this period, stretching and light training were performed with an emphasis on diaphragmatic breathing. Two months post-discharge, resistance training was commenced with attention to diaphragmatic breathing, and swimming was progressively reintroduced. The rehabilitation sessions were completed over three months, after which the patient remained free of recurrent SPM.

## Discussion

Protocols for managing SPM are scarce, particularly in athletes. The treatment regimen is up to the discretion of the physician, with conservative therapy consisting of bed rest, oxygen therapy, and analgesics generally being utilized for SPM patients [9]. Cessation of training may jeopardize an athlete’s competitive career; thus, guiding the duration of exercise restriction remains a clinical challenge.

A retrospective series of ten sports‑related cases reported that all patients were treated conservatively with analgesia, oxygen, and observation. Complete resorption occurred within three to eight days, and hospital stays ranged from two to six days. The authors concluded that restricting athletic activity after the first episode is generally unnecessary, except for scuba diving [10].

In a retrospective study of 30 adolescents, the median interval between initial and recurrent episodes was 32-315 days [11]. There is a paucity of literature on the appropriate timing for patients to resume air travel or contact sports after pneumomediastinum. However, it is recommended that patients avoid strenuous exercise and activities that increase intrathoracic pressure, such as weightlifting and scuba diving, until they are completely asymptomatic [12].

Our patient experienced three episodes of SPM over approximately seven months in the context of competitive swimming. He reported using breath‑holding techniques during training, an activity that can markedly increase intra‑alveolar pressure and predispose to the Macklin effect [2,13]. His initial episode resolved with observation and analgesics, consistent with the conservative management described above. However, in the first and second episodes, the rest period was limited to two weeks, which may have been insufficient for complete recovery. As a result, he experienced three recurrent episodes within a span of only a few months. CT imaging demonstrated mediastinal emphysema centered around the left main bronchus, suggesting persistent local tissue vulnerability. The recurrence likely resulted from incomplete healing of bronchial tissues combined with insufficient restriction of breath‑holding maneuvers. During the first two episodes, the patient gradually resumed swimming within two weeks and began rigorous breath‑hold training soon thereafter. Although the general literature suggests that rapid return to sport is safe [10], these recommendations are based on isolated episodes. They may not apply to athletes with a history of recurrence or to sports that rely heavily on breath-holding, such as competitive swimming. The third hospitalization prompted a more structured intervention. After discharge, the patient enrolled in supervised rehabilitation with a sports trainer. He avoided even land‑based training and swimming for the two months, focusing on stretching and diaphragmatic breathing; this approach aims to reduce intrathoracic pressure during exertion and limit the Valsalva maneuvers, thereby reducing shear forces on the alveoli [3,4]. Resistance training and gradual reintroduction of swimming were commenced thereafter, emphasizing controlled breathing and avoidance of breath‑holding. No recurrences were observed over a three‑month follow‑up. This outcome suggests that, for athletes with recurrent SPM, extended rest, targeted respiratory rehabilitation, and avoidance of breath-holding may be beneficial. In our swimmer, standard recommendations based on non‑athlete populations may have been insufficient; the demands of competitive swimming, with frequent underwater breath‑holding and high intrathoracic pressures during turns, likely contributed to the recurrence. Although no universal guideline exists, several diagnostic and management algorithms for SPM have been proposed, including a commonly cited adult algorithm [14] and more recent pediatric-focused proposals [15]. In clinically stable patients without features suggestive of aerodigestive injury, management is generally supportive observation, rest, analgesia, and supplemental oxygen as needed [15,16], with additional invasive testing reserved for concerning presentations. Patients are typically advised to avoid activities that markedly increase intrathoracic pressure until symptoms resolve, scuba diving is generally discouraged, and air travel is often deferred until radiographic resolution [17].

Our intervention aligns conceptually with the general advice to avoid strenuous exercise until symptoms resolve but extends the rest period and incorporates breathing retraining. A limitation of this report is that we did not perform interval imaging to confirm complete radiographic resolution between episodes, as the findings were detectable only on CT, and follow-up CT solely for this purpose was considered unjustified given the cumulative radiation exposure. Therefore, incomplete healing remains a proposed explanation rather than a proven mechanism.

## Conclusions

Although there is no high-quality evidence to support a specific duration of rest after SPM, our experience indicates that two months of restricted activity, followed by gradual return under supervision, facilitated full recovery and prevented recurrence. This timeframe allowed for the healing of bronchial microinjury and improved the athlete’s breathing technique.
